# Genomics for All: International Open Science Genomics Projects and Capacity Building in the Developing World

**DOI:** 10.3389/fgene.2019.00095

**Published:** 2019-02-15

**Authors:** Martin Hetu, Konstantia Koutouki, Yann Joly

**Affiliations:** ^1^Department of Human Genetics, Faculty of Medicine, Centre of Genomics and Policy, McGill University, Montreal, QC, Canada; ^2^Faculty of Law, Université de Montréal, Montreal, QC, Canada

**Keywords:** open science, genomics, research and development, capacity building, database, international scientific community, developing countries, genomic medicine

## Abstract

Genomic medicine applications have the potential to considerably improve health care in developing countries in the coming years. However, if developing countries do not improve their capacity for research and development (R&D) in the field, they might be left out of the genomics revolution. Large-scale and widely accessible databases for storing and analyzing genomic data are crucial tools for the advancement of genomic medicine. Building developing countries' capacity in genomics is accordingly closely linked to their involvement in international human genomics research initiatives. The purpose of this paper is to conduct a pilot study on the impact of international open science genomics projects on capacity building in R&D in developing countries. Using indicators we developed in previous work to measure the performance of international open science genomics projects, we analyse the policies and practices of four key projects in the field: the International HapMap Project, the Human Heredity and Health in Africa Initiative, the Malaria Genomic Epidemiology Network and the Structural Genomics Consortium. The results show that these projects play an important role in genomics capacity building in developing countries, but play a more limited role with regard to the potential redistribution of the benefits of research to the populations of these countries. We further suggest concrete initiatives that could facilitate the involvement of researchers from developing countries in the international genomics research community and accelerate capacity building in the developing world.

## Introduction

The establishment of large-scale genomic databases to store data generated through genome sequencing is crucial for the advancement of genomic medicine. These catalogs of genetic data have played a critical role in the discovery of thousands of genes associated with Mendelian and multifactorial diseases. Complete characterization of the genetic aspects of complex diseases however requires the identification of the whole spectrum of human genomic variations and their interactions through new research (Brown et al., 2008; Green et al., 2011). For example, researchers around the globe are now using genomic databases to identify and categorize genetic variations associated with nearly 200 types and numerous sub-types of cancer (Chin et al., 2011; Cancer Genome Atlas, 2018; International Cancer Genome Consortium, 2018). These databases play a fundamental role in enabling research and development (R&D) capacity building in genomics since they constitute research tools that are often widely accessible to researchers.

Such initiatives have resulted in major progress in human genomics and led to the development of new medical technologies currently contributing or with the potential to contribute in the future to improving health care on a global level (Singer et al., 2005). The progress made in genomic medicine has, however, mainly taken place in developed countries^1^ while advances in genomics in developing countries are perceived as out of reach. Examples of the successful implementation of large-scale genomics projects at the national level have materialized in a limited number of countries in the developing world (Seguin et al., 2008a; Manolio et al., 2015). Such projects include the establishment, in 2004, of the Mexican National Institute of Genomic Medicine to carry out an extensive genotyping project to map genomic variation within the country's population (Jimenez-Sanchez, 2003; Seguin et al., 2008b). Thailand, India and some Middle Eastern countries, such as Saudi Arabia, Qatar and Kuwait, have also launched similar national and regional genomics research programs (Indian Genome Variation Consortium, 2005; Tadmouri et al., 2006; Tongsima et al., 2008; Abu-Elmagd et al., 2015). Nevertheless, a number of experts are worried that if most developing countries do not substantially improve their capacity for R&D in genomics and integrate the international research community, this will result in a technological lag comparable to that associated with the information technology revolution (Smith et al., 2004; Isaacson, 2016; Popejoy and Fullerton, 2016; Mathew et al., 2017). In this regard, many authors have pointed out how difficult it is for researchers in developing countries to participate in large-scale international genomics research projects and to benefit from their achievements due, among other things, to lack of funding and to policies based on commercialization and intellectual property (IP) protection preventing their access to necessary knowledge (Barton, 2002; Wonkam and Mayosi, 2014; Forero et al., 2016; Helmy et al., 2016).

The purpose of this paper is to conduct a pilot study on the impact of the collaboration and innovation policies of international open science genomics projects on capacity building in genomics R&D in developing countries. Moreover, a secondary objective is to apply and test research performance indicators we presented in previous work that are designed to include broader objectives than the indicators generally used by decision-makers (Hetu et al., 2017). In order to do this, we will begin by describing the methodology used in our analytical assessment. We will then present our findings and, finally, undertake a critical discussion of the results and propose a way forward concerning some of the issues identified.

## Methods

In this section, we will first describe the international open science genomics projects that we selected for inclusion in the pilot study forming the basis of our discussion. We will then introduce the methods for the data collection and analysis.

### International Open Science Genomics Projects

The number of international open science genomics projects remains rather limited. For the purposes of our analysis, we selected four projects based on specific criteria. In the course of our selection process, we first performed a search aiming to identify all international open science genomics projects that had reached an advanced stage of their data collection activities. We then selected relevant projects based on their global human health objectives, their international character, their focus on different areas of genomics and diseases, the execution of some of their data collection and analysis activities in developing countries and the availability of the information required for analysis. It was not necessary for projects to be specifically designed to contribute to capacity building in developing countries. The four projects that we selected are the International HapMap Project (HapMap), the Human Heredity and Health in Africa Initiative (H3Africa), the Malaria Genomic Epidemiology Network (MalariaGEN) and the Structural Genomics Consortium (SGC). Table 1 presents information regarding the projects' objectives and funding sources.

**Table 1:** International open science genomics projects.

	**International HapMap Project**	**Human Heredity and Health in Africa Initiative**	**Malaria Genomic Epidemiology Network**	**Structural Genomics Consortium**
Years of operation	2002–2016	2010–Present	2005–Present	2003–Present
Objective	Map the most common human genetic variations and describe their nature, location in the DNA sequence and distribution within a given population and between populations in different places in the world.	Facilitate genomics research and research on environmental determinants of diseases in Africa in order to improve the health of African populations.	Identify the genetic specificities associated with the transmission of malaria and with resistance to malaria developed by humans.	Identify the three-dimensional structure of human proteins and parasite proteins on a large-scale and in a cost-effective manner in order to facilitate research and development of new medications.
Main countries with research institutions involved in the project	Canada, China, Japan, Nigeria, United Kingdom, and United States	Benin, Botswana, Ethiopia, Ghana, Mali, Nigeria, South Africa, Uganda, United States, and Zimbabwe	Angola, Bangladesh, Brazil, Burkina Faso, Cambodia, Cameroon, China, Colombia, Democratic Republic of the Congo, Equatorial Guinea, Gabon, Ghana, Guinea, Guinea Bissau, India, Indonesia, Ivory Coast, Kenya, Laos, Madagascar, Malaysia, Malawi, Mali, Myanmar, Nigeria, Papua New Guinea, Peru, Senegal, Sudan, Tanzania, Thailand, Uganda, United Kingdom, and Vietnam	Brazil, Canada, Germany, Sweden, United Kingdom, and United States
Main funding organizations	Chinese Academy of Sciences, Chinese Ministry of Science and Technology, George S. and Dolores Dore Eccles Foundation, Genome Canada, Genome Quebec, Hong Kong Innovation and Technology Commission, Japanese Ministry of Education, Culture, Sports, Science and Technology, Natural Science Foundation of China, SNP Consortium, United States National Institutes of Health, University Grants Committee of Hong Kong, W.M. Keck Foundation and Wellcome Trust	United States National Institutes of Health and Wellcome Trust	Bill & Melinda Gates Foundation, Foundation for the National Institutes of Health, United Kingdom Medical Research Council and Wellcome Trust	AbbVie, Bayer Pharma AG, Boehringer Ingelheim, Canada Foundation for Innovation, Eshelman Institute for Innovation, Genome Canada, Innovative Medicines Initiative, Janssen, Merck KGaA, MSD, Novartis Pharma AG, Ontario Ministry of Research, Innovation and Science, Pfizer, Sao Paulo Research Foundation, Takeda and Wellcome Trust
Website	http://hapmap.ncbi.nlm.nih.gov/	https://h3africa.org/	https://www.malariagen.net/	http://www.thesgc.org/

### Data and Analysis

At present, robust data on the capacity building performance of large-scale genomics research projects, such as data pertaining to the development of research infrastructures or training of researchers, are lacking. This situation limits decision-makers' ability to address critical issues concerning the efficiency and utility of the genomics innovation system. The limited data available generally concern IP and largely ignore the social benefits associated with collaborative research as well as the broader impact of knowledge-sharing activities on innovation (Langford et al., 2006). The main data sources used by public decision-makers include incomplete inventories of invention disclosures, patent applications, patents granted, licenses, and spin-off companies (Joly et al., 2012). Decision-makers generally do not seem to consider the exchange of knowledge among different actors of the innovation system as well as capacity building to be independently valuable objectives of scientific research projects.

This issue is accentuated in developing countries, which have to cope with a crucial lack of cutting-edge technology research infrastructures, little investment in research and a limited pool of researchers with appropriate training to develop and carry out research projects in an independent manner (United Nations Millenium Project Science Technology Task Force Genomics Working Group, 2004; Forero et al., 2016; Helmy et al., 2016). In this context, a broad understanding of the concept of research valorisation provides a more inclusive theoretical framework for the assessment of the performance of research projects. Valorisation is a broad concept encompassing all channels that contribute to ensuring that the outcomes of scientific knowledge add value beyond the scientific domain. It is a process of realization of relevant added value products in a given domain for broad societal benefit. It puts forward the idea that the importance of both economic and social values should be recognized. Valorisation is thus broader than commercialization, which is motivated primarily by profit (Slaughter and Leslie, 1997, 2001; Bridgman and Wilmott, 2007; Benneworth and Jongbloed, 2010; Lal et al., 2011; Joly et al., 2012).

We use indicators inspired by the research valorisation concept that we developed and presented in a previous paper to assess the benefits of international open science genomics projects with respect to R&D capacity building in genomics and access to genomic medicine applications in developing countries (Hetu et al., 2017). As capacity building is an activity that is difficult to measure in a consistent manner through publicly available metrics or with the help of metrics that might be partially available upon request, these indicators do not aim to provide a comprehensive view of the impact of genomics projects on this regard. The methodology we used to develop the indicators is described in detail in our previous paper (Hetu et al., 2017). The indicators are presented in Table 2.

**Table 2:** Indicators: Measuring international open science genomics projects' performance in fostering genomic capacity building in the developing world.

Indicator no. 1	The data collected in the framework of the project include data collected from populations in developing countries.
Indicator no. 2	Part of the project concerns a disease significantly affecting the health of populations in developing countries.
Indicator no. 3	Researchers in developing countries are involved in the project.
Indicator no. 4	Researchers in developing countries have access to the data collected in the context of the project.
Indicator no. 5	The data collected in the context of the project are used by researchers in developing countries.
Indicator no. 6	The project contributes to the development of research infrastructures in developing countries.
Indicator no. 7	Decision-making positions are assigned to researchers and managers in developing countries.
Indicator no. 8	The project includes training opportunities accessible and relevant to researchers/students in developing countries.
Indicator no. 9	The project's intellectual property management policies are favorable to developing countries.

Our analysis, executed in October 2017, involved the consultation of policy documents related to innovation and research valorisation, published descriptive articles and the websites of the four selected projects. The HapMap project was terminated in June 2016. In order to be able to retrieve relevant information from its website, we used the Internet Archive's Wayback Machine to access the version of the website that was online on March 31st 2016. For the purposes of this study, policy documents are defined as official documents concerning, among other things, access to data, IP management, publication of research findings, consent to research, terms governing the citation of data sources, funding, and the involvement of the communities concerned by the research. We identified six policy documents on HapMap' website, 11 documents on H3Africa's website, 12 documents on MalariaGEN's website, and no document on SGC's website. We also performed a literature review in order to find articles describing the projects^2^. In addition, we contacted representatives in charge of the four projects to ask for access to policy documents not available on their websites and documents concerning research outcomes and the general administration of the projects, such as patents granted, publications by member researchers and requests for access to data. None of the projects provided any additional document. Coding of the collected material based on indicators no. 1–4 and 5–9 was performed by the first author.

Regarding indicator no. 5 concerning the use by researchers in developing countries of the data collected in the context of the projects, we performed our analysis by searching for citations of the selected projects using Web of Science. The search in Web of Science (All Databases) was performed on August 25th 2017 using these keywords: “HapMap”; “H3Africa” OR “Human Heredity and Health in Africa”; “MalariaGEN” OR “Malaria Genomic Epidemiology Network”; “Structural Genomics Consortium.” Duplicates were withdrawn from the search results. Our search identified 3,185 publications citing HapMap, 43 publications citing H3Africa, 18 publications citing MalariaGEN, and 143 publications citing SGC. All publications were manually coded by the first author according to this code: (1) involves at least one researcher from a developing country; (2) does not involve any researcher from a developing country; (3) does not consist in an original genomics research study related to human health. The publications that were not original genomics research studies related to human health were excluded from the analysis.

## Results

The findings of our pilot study on the impact of international open genomics projects on capacity building in the developing world are presented in Table 3^3^.

**Table 3:** A first look at the impact of international open science genomics projects on capacity building in the developing world.

	**International HapMap Project**	**Human Heredity and Health in Africa Initiative**	**Malaria Genomic Epidemiology Network**	**Structural Genomics Consortium**
Indicator no. 1: The data collected in the framework of the project include data collected from populations in developing countries	Seventy-eight percent (78%) of the data were collected from populations in developing countries.	One hundred percent (100%) of the data were collected from populations in developing countries.	One hundred percent (100%) of the data were collected from populations in developing countries.	The source of the data is not systematically specified.
Indicator no. 2: Part of the project concerns a disease significantly affecting the health of populations in developing countries	The data are relevant for research on diseases affecting the population of developing countries.	The data are relevant for research on diseases affecting the population of developing countries.	The data are relevant for research on diseases affecting the population of developing countries.	The data are relevant for research on diseases affecting the population of developing countries.
Indicator no. 3: Researchers in developing countries are involved in the project	Twenty-eight percent (28%) of the research centers involved were located in developing countries.	The majority of the research centers involved were located in developing countries.	Fifty-one percent (51%) of the research centers involved were located in developing countries.	Seventeen percent (17%) of the main research centers involved were located in developing countries.
Indicator no. 4: Researchers in developing countries have access to the data collected in the context of the project	Data are placed in the public domain and are accessible to all.	Data are accessible to all after the expiration of temporary measures favoring the researchers who collected the data.	Data are accessible to all after the expiration of temporary measures favoring the researchers who collected the data.	Data are placed in the public domain and are accessible to all.
Indicator no. 5: The data collected in the context of the project are used by researchers in developing countries	Twenty-seven percent (27%) of the 2,057 published studies using data from the project involved researchers from developing countries.	One hundred percent (100%) of the three published studies using data from the project involved researchers from developing countries.	Eighty-nine percent (89%) of the nine published studies using data from the project involved researchers from developing countries.	Twenty percent (20%) of the five published studies using data from the project involved researchers from developing countries.
Indicator no. 6: The project contributes to the development of research infrastructures in developing countries	No specific program addressing the development of research infrastructures.	The project contributed to the establishment of genomics research centers and a bioinformatics network.	Impact on the development of research infrastructures in developing countries cannot be clearly identified.	Impact on the development of research infrastructures in developing countries cannot be clearly identified.
Indicator no. 7: Decision-making positions are assigned to researchers and managers in developing countries	None of the project's decision-making positions were attributed to researchers from developing countries.	Forty-four percent (44%) of the project's decision-making positions were attributed to researchers from developing countries.	Sixty-three percent (63%) of the project's decision-making positions were attributed to researchers from developing countries.	One percent (1%) of the project's decision-making positions was attributed to researchers from developing countries.
Indicator no. 8: The project includes training opportunities accessible and relevant to researchers/students in developing countries	No specific training program accessible to researchers/students in developing countries.	All of the research sub-projects must involve a training component and training workshops are frequently organized.	The project involves training scholarships and training workshops are frequently organized.	The project involves training scholarships, but they are not accessible to researchers/students in developing countries. Training workshops are frequently organized.
Indicator no. 9: The project's intellectual property management policies are favorable to developing countries	Discoveries resulting from research using the project's data may be patented. No policy favoring the redistribution of benefits from patented discoveries to populations of developing countries.	Discoveries resulting from research using the project's data may be patented. No policy favoring the redistribution of benefits from patented discoveries to populations of developing countries.	Discoveries resulting from research using the project's data may not be patented, except if IP protection is necessary for the transfer of technology to developing countries. Benefits from patented discoveries must be redistributed to populations of developing countries.	Discoveries resulting from research using the project's data may be patented. No policy favoring the redistribution of benefits from patented discoveries to populations of developing countries.

## Discussion

Our analysis of indicators no. 1 and 2 shows that the four selected projects are concerned with the health of populations in developing countries. Findings for indicator no. 1 show that the projects collecting human genetic data gathered a significant portion of their data from populations in developing countries. H3Africa and MalariaGEN were specifically designed to address health problems afflicting populations of developing countries and predictably performed well. While SGC does not specifically focus on developing countries, it nevertheless launched a program in collaboration with major non-governmental organizations to address health problems specific to populations in developing countries. Analysis of indicator no. 2 shows that all projects address health issues that are prevalent in developing countries.

The members of the research community involved in these four international genomics projects seem to have made considerable efforts to orient genomics research toward problems that are relevant to the health of populations in developing countries. However, these efforts still remain insufficient to make up for developing countries' significant lag in genomics research given their demographic importance and their underrepresentation in genome-wide association studies (Hindorff et al., 2018). In 2011, 96% of genome-wide association studies targeted populations of European ancestry, generally located in developed countries in North America and Europe (Bustamante et al., 2011). A recently updated study published in 2016 found that 20% of genome wide association studies now involved participants from non-European populations, generally found in, or originating from developing countries in Africa, Asia, Latin America and the Middle East (Popejoy and Fullerton, 2016). The increase in percentage was, however, mainly due to research projects involving Asian populations from Japan and South Korea, which are developed countries, as well as China and India. The low level of genomics research nevertheless remained a considerable problem and saw very little progress in other areas of the developing world. Yet, it has been established that the frequency of genetic variants contributing to disease can differ from one population to the next, as can be concluded from the high prevalence of sickle-cell disease, thalassemia and glucose-6-phosphate dehydrogenase deficiency in specific African communities (Rotimi and Jorde, 2012). Due to the variation in frequency of rare genetic variants across populations, a lack of diversity in genetic studies is likely to skew the medical community's understanding of which variants are important (Bustamante et al., 2011). Moreover, the generalization of the importance of specific variants to most populations despite their different ancestry may lead to misclassification of benign variants as pathogenic and introduces considerable risks of misdiagnosis in certain populations. Clinical guidelines now suggest that ancestry-matched controls should be used to interpret variants (MacArthur et al., 2014; Richards et al., 2015). Accordingly, further efforts are required to collect genomic data on underrepresented populations in developing countries in order to evaluate the pathogenicity of novel variants and re-evaluate known variants (Manrai et al., 2016; Rotimi et al., 2017). The international genomics research community should pursue initiatives aiming to include populations of developing countries in projects involving data collection. This could be executed through stricter requirements in favor of inclusion of diverse populations in grant programs (Mathew et al., 2017).

Our findings resulting from the analysis of indicators no. 3–5 suggest that some international open science genomics projects fostered the integration of genomics researchers from developing countries in the international genomics research community and favored their access to the data generated in the context of such projects. Analysis of indicator no. 3 indicates that researchers and research centers in developing countries have been significantly involved in the formation of HapMap, H3Africa, and MalariaGEN. The SGC project is mainly based in developed countries, but nevertheless involves the participation of a research institution located in a developing country. Analysis of indicator no. 4 shows that the selected projects opted for knowledge management mechanisms favoring access by researchers in developing countries to the genetic data necessary for their research. HapMap and SGC's data are placed in the public domain, while researchers from developing countries are given priority access to H3Africa and MalariaGEN's data before they are made accessible to all researchers. Analysis of indicator no. 5 reveals that use of the data from the H3Africa, MalariaGEN and SGC projects remains limited. The comparatively frequent use of HapMap's data by researchers in developing countries is however encouraging since it indicates the willingness as well as the capacity of researchers in developing countries to use data from international open science genomics projects.

International genomics research projects have the potential to play an important role in the transfer of implicit knowledge, such as the expertise of experienced researchers, as well as explicit knowledge, such as genetic data, to researchers in the developing world. Indeed, the considerable use of HapMap data by international researchers reveals that international open science genomics projects meet a need for genomic data of researchers based in developing countries. These limited findings bode well for the future use of more recently launched international genomics research projects, such as H3Africa and MalariaGEN. The integration of developing countries' research institutions in international genomics projects should be further pursued in order to increase collaboration between researchers located in developing and developed countries as well as to frame the research agenda within the international community toward objectives that will further benefit developing countries (Forero et al., 2016). International organizations operating at the intersection of science and policy, such as the World Health Organization, could help in this regard by playing a supervisory role and facilitating the discussion between different stakeholders of the genomics research community (Chen and Pang, 2015; Antonarakis, 2017).

On the other hand, analysis of indicators no. 6–8 shows that the impact of the projects on the development of research infrastructures and the training of researchers in developing countries has overall remained limited. Due to their focus on developing countries, H3Africa and MalariaGEN's influence has foreseeably been much more significant in this respect than that of the HapMap and SGC projects. Analysis of indicator no. 6 illustrates the significant contribution of H3Africa in the development of genomic databases and of a bioinformatics network on the African continent. Analysis of indicator no. 7 however shows that decision-making positions have been given to researchers in developing countries mainly by H3Africa and MalariaGEN, which are specifically focusing on developing countries. Among dozens of identified decision-making positions, HapMap and SGC together assigned a single one of these to a researcher from a developing country. Analysis of indicator no. 8 displays again that H3Africa and MalariaGEN contributed to the training of researchers in developing countries while HapMap and SGC had a limited impact in this regard.

These results reveal that the impact of international open science genomics projects on the development of research infrastructures and the training of researchers from developing countries depends largely on the predefined role of the project. While all of the selected projects seem to contribute to providing access to raw data to researchers in developing countries through open knowledge sharing policies, only H3Africa and MalariaGEN seem to make significant contributions to the acquisition by developing countries of the equipment and expertise needed to use human genomic data for R&D activities. In this regard, the research infrastructures developed in the context of H3Africa could eventually contribute to the establishment of an independent genomics innovation system on the African continent. Equipped with the laboratories, databases, and bioinformatics systems required to conduct complex genomics research, researchers in African countries should be better able to seek external funding for their projects. Moreover, the training programs set up through H3Africa and MalariaGEN constitute powerful mechanisms for implicit knowledge transfer, helping researchers in developing countries acquire the expertise in medical genomics and project management required to set up their own research initiatives.

Infrastructure creation and training initiatives targeting developing countries do not seem to have been implemented in many international projects in genomics. As these initiatives play a key role in the development of research capacity in developing countries and facilitate their participation in the international genomics research community, members of this community should push for the adoption of such initiatives in existing and future projects. These projects could, for example, contribute to the establishment of centers of excellence in genomics advancing sequencing capacities in countries where genomic data are collected (Sirisena and Dissanayake, 2017). These centers could then be used to offer training in genomics and bioinformatics to local researchers, clinicians, and policy-makers as well as provide the foundations for the development of independent local databases and bioinformatics networks (Patrinos et al., 2011; Mathew et al., 2017). The experience of the H3Africa project could provide a useful baseline in this regard.

Finally, analysis of indicator no. 9 shows that all of the selected projects established measures to ensure that access to the data that they generated would not be blocked by IP issues. These results are in accordance with the view that strong IP protection of new research data is not desirable with regard to the mitigated success of the present biomedical innovation system in terms of development of new therapeutic applications (Gold et al., 2010; Williams, 2013; Ward et al., 2014). However, only MalariaGEN has established measures providing specifically for the redistribution of the benefits of R&D leading to new therapeutic applications to populations in developing countries. Indeed, IP management policies designed to favor populations of the developing world and facilitate their access to new therapeutic applications, such as the imposition of advantageous patent licensing policies, generally do not seem to have been adopted by international open science genomics projects.

These projects could, however, institute IP management policies setting limits on the geographical zones where patents can be obtained for an innovation resulting from research performed using their data or requiring that the owner of a patent license provide access or set different prices for developing countries (Guebert and Bubela, 2014). Measures of this type would have the effect of facilitating the redistribution of benefits to developing countries while maintaining R&D incentives for Western companies. In addition, international projects in genomics should make sure that the discoveries they generate are used for the benefit of populations in developing countries as quickly as possible. For example, they should establish policies facilitating the transfer of technologies developed in the context of the project to the private sector in order to accelerate the development and commercialization of new therapeutic applications while, at the same time, providing for equitable accessibility to these applications. They should also create ties with public health bodies to ensure that their discoveries are integrated into public health policies.

## Conclusion

Large-scale genomic databases constitute critical research tools for scientific progress in the field of genomics. In order for developing countries to benefit from the genomics revolution, they need to improve their R&D capacity and further integrate the international genomics research community. Our findings showed that international open science genomics projects play an important role in genomics capacity building in developing countries. For example, the research performed within these projects proved to be relevant to the health of populations of developing countries and favorable to the integration of researchers from developing countries into the international genomics research community. However, the projects' impact on the development of research infrastructures in developing countries and on the training of local researchers is generally positive, but remains unequal as some of the selected projects have a significantly stronger impact than others in this regard. Finally, the results of our study showed that international open science genomics projects seem to play a limited role in facilitating the redistribution of the potential benefits of research in developing countries.

Building on international open science genomics projects' strengths and weaknesses underlined in this paper, the international genomics research community has the opportunity to step in to accelerate genomics capacity building in developing countries and ensure that their populations benefit from advances in genomic medicine. Potential useful initiatives include allocating funding to local genomic data collection efforts in developing countries to support R&D capacity building and participation in international initiatives. In this regard, funding offered to genomics research projects by local national research agencies, such as the South African National Research Foundation, as well as funding from agencies in developed countries, such as the Wellcome Trust and the United States National Institutes of Health, played an important role in the development and strengthening of genomics research capacity on the African continent (Helmy et al., 2016; Mulder et al., 2018).

Finally, this pilot study tested a set of research performance indicators selected with the specific goal of measuring the impact of large-scale genomics projects in the developing world. We demonstrated that the use of broad indicators based on the achievement of social and economic objectives is more likely to provide exhaustive, accurate, and useful data for decision-makers regarding the equitable and inclusive performance of such projects.

## Author Contributions

MH devised the methodology, collected and analyzed the data, and drafted the paper. KK and YJ devised the methodology, revised the results, and assisted with the drafting of the paper.

### Conflict of Interest Statement

The authors declare that the research was conducted in the absence of any commercial or financial relationships that could be construed as a potential conflict of interest.

## Footnotes

^1^Developed countries are defined as the 39 countries in the list of advanced countries and developing countries are defined as the 153 countries in the list of emerging market and developing economies published annually by the International Monetary Fund (International Monetary Fund, 2017).

^2^The relevant policy documents, articles and sections of the projects' websites used in the analysis are listed in the References section of the Supplementary Material document (Table S1).

^3^Detailed results are provided in the Supplementary Material document (Table S1).
