# Supplementary material for: Genomics for All: International Open Science Genomics Projects and Capacity Building in the Developing World
Source: Front Genet. 2019 Feb 15;10:95. doi: 10.3389/fgene.2019.00095 (PMC6384230; doi:10.3389/fgene.2019.00095)
Supplement: Supplementary file 1 [file Table_1.docx]

***Supplementary Material***

**Genomics for All: International Open Science Genomics Projects and Capacity Building in the Developing World**

Martin Hetu*, Konstantia Koutouki & Yann Joly

* Correspondence:

Martin Hetu: martin.hetu@mail.mcgill.ca

**Table S1. A first look at the impact of international open science genomics projects on capacity building in the developing world: Detailed results**

|  | **International HapMap Project** | **Human Heredity and Health in Africa Initiative** | **Malaria Genomic Epidemiology Network** | **Structural Genomics Consortium** |
| --- | --- | --- | --- | --- |
| **Indicator No. 1: The data collected in the framework of the project include data collected from populations in developing countries** | In the initial phase of the project, samples were taken from 270 people, including 90 Nigerians and 45 Chinese people. The other samples were taken from residents of the United States with origins in Northern and Western Europe as well as from residents of Japan (1). In the initial phase of the project, 50% of the data was therefore collected from people in developing countries.  In a following phase of the project, new samples were collected. The seven target populations were the Maasai community in Kenya (205 samples), the Luhya community in Kenya (122 samples), the Chinese community in the United States (129 samples), the Gujarati Indian community in the United States (117 samples), the Tuscan Italian community in the United States (117 samples), the African-American community in the United States (106 samples) and the Mexican community in the United States (104 samples) (2). Thus, in this phase of the project, 87% of the data collected came from communities with origins in developing countries since the samples were collected from populations in a developing country (36% of the samples) or from people living in the United States, but originating in a developing country (51% of the samples). | The project plans to gather samples from 50 000 to 75 000 people. The samples will be collected uniquely from populations of African countries by the research centres participating in the project (8, 9). All of the data collected for the project will thus be taken from populations of developing countries. | The project studies the genomes of humans and of parasites responsible for malaria as well as mosquitos that transmit it. The samples for the study of the human genome were gathered from members of the populations of 32 developing countries: (23). All of the data collected from humans thus comes from populations in developing countries. | The project does not systematically provide information on the source of the data gathered. |
| **Indicator No. 2: Part of the project concerns a disease significantly affecting the health of populations in developing countries** | The goal of the project was to gather data to produce a map of human haploids that could be used by researchers in genomic medicine for their own research projects on specific diseases. Some of these data could be relevant to research on diseases that are prevalent in developing countries. | All of the project’s activities concern diseases affecting the populations of African countries, such as tuberculosis and trypanosomiasis. African countries are all developing countries (9). | The research done in the framework of the project only concerns malaria, a disease that mainly affects developing countries (24). | The project does not uniquely concern diseases mainly affecting developing countries. However, much of the research activities are devoted to such diseases. For example, the project revealed the proteins of parasites responsible for malaria, Chagas disease, African sleeping sickness and leishmaniosis (43). The project has also set up the Structure-guided Drug Discovery Coalition in partnership with the Medicine for Malaria Venture and the Global Alliance for TB Drug Development, the objective of which is to identify and develop chemical assets to accelerate the R&D of drugs to treat malaria and tuberculosis (44). |
| **Indicator No. 3: Researchers in developing countries are involved in the project** | Twenty-eight (28) research centres were involved in gathering and processing the data in the framework of the project. Seven of the research centres were located in China and one in Nigeria. The other research centres were located in the United States, Japan, Canada and the United Kingdom (3). Twenty-eight percent (28%) of the research centres involved were thus located in developing countries. | As of 2016, 26 research groups in nearly 27 African countries are involved in the project. Research centres in Canada, Belgium, France, the United States and the United Kingdom are also involved. Their number is, however, limited compared with the number of research centres located in developing countries (10-12). The great majority of the research centres involved in the project thus come from developing countries. | There are 81 different research centres involved in the project. They include 41 research centres located in developing countries and 40 research centres located in developed countries (25). Therefore, 51% of the research centres involved in the project are located in developing countries. | The main research centres involved in the project are affiliated to the University of Toronto in Canada, Oxford University in the United Kingdom, the University of Campinas in Brazil, Karolinska Institute in Sweden, the University of North Carolina at Chapel Hill in the United States and Goethe University Frankfurt in Germany (45). The principal research and funding partners in the project include nine Western pharmaceutical companies and five funding institutions from Canada, Brazil and the United Kingdom (46). Accordingly, researchers from one developing country (Brazil) are involved in the project. Moreover, the project maintains over 250 research relationships with academic and private industry researchers in various countries, but the information on these collaborations is not systematically reported (47, 48). |
| **Indicator No. 4: Researchers in developing countries have access to the data collected in the context of the project** | All of the data generated in the context of the project were placed in the public domain (4, 5). All researchers in developing countries therefore have access to the data gathered during the project. | Initially, after the data are integrated into the H3ABioNet database, a pan-African bioinformatics network developed to support H3Africa’s research activities, the project gives nine months of priority access to the member researchers who collected the data. Subsequent access to the data is available to all researchers following approval of their access request by the project’s data access committee, but involves a 12-month embargo on publications so as to protect the publication rights of the researchers who collected the data. After that period, the data are available to all researchers following approval of their access request by the project’s data access committee (13, 14). Researchers in developing countries are thus given priority access to the data gathered in the context of the project. | The project’s data sharing policies are based on the principles described in the Fort Lauderdale Agreement, the outcome of a meeting of genomic scientists and professionals held in 2003. Access to the human genetic data collected through the project is provided to all researchers nine months after they have been collected, but entails a publication embargo until publication of the initial analysis of the data by the project member researchers who collected the data (26-31). The genetic data on the parasites responsible for malaria are also shared in accordance with the Fort Lauderdale Agreement. The sharing of data on parasites entailed a publication embargo until publication of the initial analyses by the project-member researchers who collected the data. The publications restrictions have now been lifted on all parasites datasets since February 2017 (32-34). The genetic data on mosquitos that transmit malaria are available to all researchers, but subject to a publication embargo until publication of the initial analyses by the researchers who collected the data (34). Participating researchers in developing countries therefore have priority access to the human genetic, parasites and mosquitos data collected in the framework of the project. | All of the data produced by the project are placed in the public domain and are accessible to all researchers at the same time (49). All researchers in developing countries therefore have access to the data gathered in the context of the project. |
| **Indicator No. 5: The data collected in the context of the project are used by researchers in developing countries** | Two thousand fifty-seven (2 057) articles (out of a total of 3 185 found through our research) describe studies executed using data generated by the project. Five hundred and fifty-seven (557) of those studies involved researchers from developing countries. Twenty-seven percent (27%) of the 2 057 published studies using data from the project involved researchers from developing countries. | Three articles (out of a total of 43 found through our research) describe studies executed using data generated by the project. The three studies involved researchers from developing countries. One hundred percent (100%) of the three published studies using data from the project involved researchers from developing countries. | Nine articles (out of a total of 18 articles found through our research) described studies executed using data generated by the project. Eight of those studies involved the participation of researchers from developing countries. Eighty-nine percent (89%) of the nine published studies using data from the project involved researchers from developing countries. | Five articles (out of a total of 143 articles found through our research) described studies executed using data generated by the project. One of the studies involved the participation of researchers from developing countries. Twenty percent (20%) of the five published studies using data from the project involved researchers from developing countries. |
| **Indicator No. 6: The project contributes to the development of research infrastructures in developing countries** | The project did not include any program specifically addressing the development of research infrastructure. | The project has contributed to the development of genetic databases in South Africa, Nigeria and Uganda. The project has also contributed to the development of the H3ABioNet bioinformatics network, the purpose of which is to develop computer infrastructure so that H3Africa researchers can store, interpret and share the data that they collect. Indeed, one of the main goals for the allocation of funding to this project is to establish new research centres in genomic medicine (10, 11, 15, 16). | The project has developed a resource centre to assist researchers in their research, but both of the institutions hosting it are located in the United Kingdom (35). The project also offered support to external research projects with activities based in Africa, but their impact on the development of research infrastructures is not clear (36). | The funding for the project has probably contributed to the development of research infrastructure at the University of Toronto in Canada, Oxford University in the United Kingdom, the University of Campinas in Brazil, Karolinska Institute in Sweden, the University of North Carolina at Chapel Hill in the United States and Goethe University Frankfurt in Germany (45). Accordingly, the project probably contributed to the development of the research infrastructure in one developing country (Brazil). However, the project does not include any program specifically addressing the development of research infrastructure in developing countries. |
| **Indicator No. 7: Decision-making positions are assigned to researchers and managers in developing countries** | Two groups of researchers and managers participated in the set-up and management of the project. The first group focused on issues related to population and on the project’s ethical, legal and social implications. That group was composed of 20 researchers and managers, none of whom came from a developing country. The second group focused on methodological issues related to the project. It was composed of 23 researchers and managers, none of whom came from a developing country (6). Thus, none of the project’s decision-making positions was attributed to researchers from developing countries. | Two founding working groups set up the project. The first group focuses on questions related to non-contagious diseases and is made up of 12 researchers and managers, including five researchers and managers from developing countries. The second group studies issues related to contagious diseases and is made up of 13 researchers, including nine researchers and managers from developing countries (17). Therefore, 56 % of the decision-making positions in the founding working groups are given to researchers and managers from developing countries.  A Steering Committee and ten working groups have also been established to manage the project. The lists of researchers and managers on the committee and in the groups are not public (18).  An Independent Expert Committee was formed to advise the institutions funding the project. It is composed of 11 researchers and managers, including two from developing countries (19). Therefore, 18% of the decision-making positions on the Independent Expert Committee have been given to researchers and managers from developing countries. | The Governance Committee, responsible for the oversight of the consortium’s main projects, is made up of eight researchers and managers, five of whom are from developing countries (37). Therefore, 63% of the project’s decision-making positions have been given to researchers and managers from developing countries. | The project’s governance is managed by multiple bodies: SGC Board (Board of Directors, Board Observers, SGC Management Team), Chemical Probes (Scientific Committee, Joint Management Committee), Structure and TEP (Joint Management Committee, TEP Evaluation Group), Tissue Platforms (Oncology Joint Management Committee, Inflammation Joint Management Committee, Neurobiology Joint Management Committee), Target Prioritization Networks (Cancer, Metabolic Disease, Neurobiology). Together, these bodies are composed of 201 researchers or managers. Among these researchers or managers, only three are from developing countries (50). Thus, 1% of the decision-making positions have been allocated to researchers or managers from developing countries. |
| **Indicator No. 8: The project includes training opportunities accessible and relevant to researchers/students in developing countries** | The lack of data on this in the context of the HapMap project suggests that it did not provide for any training program. | Training researchers in developing countries is one of the project’s primary objectives. All of the research sub-projects funded in the context of the project must include training of involved researchers (10, 11, 15, 20).  Researchers who are members of the project also have access to training workshops. Seventeen (17) training workshops have been held since 2011 (21, 22). | The project has a training program for researchers in developing countries. The program has two components. The first provided bursaries associated with management of the data collected in the framework of the project for researchers located in developing countries between 2006 and 2010. Twenty (20) researchers located in 15 different developing countries received such bursaries. The second component provides for the holding of training workshops available to researchers from developing countries. At least 41 training workshops accessible to researchers from developing countries have already been held in the context of the project (38-41). | The program has established a training program that makes it possible for graduate students and post-doctoral researchers to participate in the research projects at the lab at the University of Toronto and in the laboratories of certain pharmaceutical companies. However, the training program is available only to students and post-docs studying in four Canadian universities. Twenty-five (25) researchers have participated in the program since its launch (51).  The project also participates in holding symposiums designed to share the knowledge developed in the context of the project and provide training. Twenty-nine (29) symposiums have been held since 2007 (52). |
| **Indicator No. 9: The project’s intellectual property management policies are favorable to developing countries** | The data produced by the project were placed in the public domain and, therefore, third parties should not be able to patent the data for themselves as public knowledge is generally not considered patentable in IP law due to the patentability criterion that requires patentable inventions to be novel (7). Initially, the project also required researchers requesting access to the data to refrain from acting in a manner that would prevent third parties from having access to the data, which included patenting the data. The terms of use of the data were based on copyleft licencing. However, the project encouraged the development of patentable therapeutic applications invented by using its data and the project’s IP management policies did not stipulate the redistribution of profits made from the research to populations in developing countries or the use of such profits to benefit such populations (4, 5). | Access to the data is subject to the condition of not preventing third parties from having access to the data, particularly via patents. However, the project encourages commercialisation of therapeutic applications developed using the project’s data, including patented products. The project’s IP management policies do not, however, include provisions requiring the redistribution of profits stemming from the research to the people of developing countries or the use of such profits to benefit such populations (13). | The project makes it compulsory to place all of the results of research conducted using project data in the public domain through the publication of the findings, except if IP protection of the rights over the findings is necessary for the transfer of technology to developing countries. IP rights over the results can be sought when: (1) the discovery is directly relevant to a clinical application (diagnostic, medication or vaccine); (2) the imminent granting of licences is highly probable; and (3) it has been established that IP protection is necessary to foster the development of the clinical application. The project also stipulates that if profit is made through granting licences, it must be redistributed among the participating communities and not to the researchers. Lastly, the patent licences must be ceded to non-profit organisations except when a for-profit company is able to allocate significantly more resources to the development of the clinical application. In such a case, measures must be taken to ensure global access to the clinical application (27, 29, 31, 42). | All of the data are placed directly in the public domain. The IP management policies of the project also provide that patents cannot be obtained on the results of research done in the context of the project. However, the project encourages the use of data produced in the project framework to develop patentable therapeutic applications (49, 53, 54). |

**References**

1. International HapMap Project [Internet]. Bethesda: International HapMap Project [cited 2017 Oct 11]. Which Populations Are Being Sampled; [about 2 screens]. Available from: <https://web.archive.org/web/20160427113200/http://hapmap.ncbi.nlm.nih.gov/hapmappopulations.html>.

International HapMap Project. (n.d.). Program Overview. Accessed October 4, 2018. http://cancergenome.nih.gov/abouttcga/overview.

1. Coriell Institute for Medical Research [Internet]. Camden: Coriell Institute for Medical Research; c2018 [cited 2017 Oct 11]. HapMap Project; [about 2 screens]. Available from: <https://catalog.coriell.org/1/NHGRI/Collections/HapMap-Collections/HapMap-Project>.
2. International HapMap Project [Internet]. Bethesda: International HapMap Project [cited 2017 Oct 11]. International HapMap Project; [about 2 screens]. Available from: <https://web.archive.org/web/20160420131945/http://hapmap.ncbi.nlm.nih.gov>.
3. International HapMap Project [Internet]. Bethesda: International HapMap Project [cited 2017 Oct 11]. Data Release Policy; [about 2 screens]. Available from: <https://web.archive.org/web/20160419000629/http://hapmap.ncbi.nlm.nih.gov/datareleasepolicy.html>.
4. International HapMap Project [Internet]. Bethesda: International HapMap Project [cited 2017 Oct 11]. Data Access Policy for the International HapMap Project [No longer required]; [about 2 screens]. Available from: <https://web.archive.org/web/20160520190647/http://hapmap.ncbi.nlm.nih.gov:80/abouthapmap.html>.
5. International HapMap Project [Internet]. Bethesda: International HapMap Project [cited 2017 Oct 11]. Initial Planning Groups; [about 2 screens]. Available from: <https://web.archive.org/web/20160427113142/http://hapmap.ncbi.nlm.nih.gov/initial_planning.html>.
6. Lemley M. Point of Novelty. Northwestern Law Review 2001:105;1253-1280.
7. Peterson J [Internet]. Bethesda: Human Heredity and Health in Africa Initiative; c2013 [cited 2017 Oct 10]. Project/Program Updates: H3Africa Initiative [video]. Available from: <https://www.youtube.com/watch?v=YoO6gmvHR-0>.
8. Human Heredity and Health in Africa Initiative [Internet]. Bethesda: Human Heredity and Health in Africa Initiative; c2013 [cited 2017 Oct 10]. Projects; [about 5 screens]. Available from: <http://h3africa.org/consortium/projects>.
9. Rotimi CN. H3Africa: Human Heredity and Health in Africa. Bethesda: National Human Genome Research Institute; 2014.
10. H3Africa Consortium; Rotimi C, Abayomi A, Abimiku A, Adabayeri VM, Adebamowo C, et al. Enabling the genomic revolution in Africa: H3Africa is developing capacity for health-related genomics research in Africa. Science. 2014;344:1346-1348.
11. Ramsay M, Sankoh O. African partnerships through the H3Africa Consortium bring a genomic dimension to longitudinal population studies on the continent. Int J Epidemiol. 2016;45:305-308.
12. Human Heredity and Health in Africa Initiative. H3Africa Consortium Data Sharing, Access and Release Policy. Bethesda: Human Heredity and Health in Africa Initiative; 2014.
13. Human Heredity and Health in Africa Initiative. Publications Policy. Bethesda: Human Heredity and Health in Africa Initiative; 2014.
14. H3Africa Working Group. White Paper – Harnessing Genomic Technologies Toward Improving Health in Africa: Opportunities and Challenges. Bethesda: H3Africa Working Group; 2011.
15. Mulder NJ, Adebiyi E, Alami R, Benkahla A, Brandful J, Doumbia S, et al. H3ABioNet, a sustainable pan-African bioinformatics network for human heredity and health in Africa. Genome Res. 2016;26:271-277.
16. Human Heredity and Health in Africa Initiative [Internet]. Bethesda: Human Heredity and Health in Africa Initiative; c2013 [cited 2017 Oct 10]. Founding Working Groups; [about 3 screens]. Available from: <http://h3africa.org/about/working-groups-main>.
17. Human Heredity and Health in Africa Initiative [Internet]. Bethesda: Human Heredity and Health in Africa Initiative; c2013 [cited 2017 Oct 10]. Working Groups; [about 2 screens]. Available from: <http://h3africa.org/consortium/working-groups>.
18. Human Heredity and Health in Africa Initiative [Internet]. Bethesda: Human Heredity and Health in Africa Initiative; c2013 [cited 2017 Oct 10]. Independent Expert Committee; [about 3 screens]. Available from: <http://h3africa.org/consortium/independent-expert-committee>.
19. Bishop OT, Adebiyi EF, Alzohairy AM, Everett D, Ghedira K, Ghouila A, et al. Bioinformatics Education – Perspectives and Challenges out of Africa. Brief Bioinform. 2015;16:355-364.
20. Human Heredity and Health in Africa Initiative [Internet]. Bethesda: Human Heredity and Health in Africa Initiative; c2013 [cited 2017 Oct 10]. Training; [about 2 screens]. Available from: <http://h3africa.org/links/resources>.
21. Human Heredity and Health in Africa Initiative [Internet]. Bethesda: Human Heredity and Health in Africa Initiative; c2013 [cited 2017 Oct 10]. Past Training Events; [about 2 screens]. Available from: <http://h3africa.org/links/resources/19-links/163-past-training-events>.
22. Malaria Genomic Epidemiology Network [Internet]. Oxford: Malaria Genomic Epidemiology Network; c2018 [cited 2017 Oct 11]. Data; [about 3 screens]. Available from: <https://www.malariagen.net/data>.
23. Malaria Genomic Epidemiology Network [Internet]. Oxford: Malaria Genomic Epidemiology Network; c2018 [cited 2017 Oct 11]. About; [about 3 screens]. Available from: <http://www.malariagen.net/about>.
24. Malaria Genomic Epidemiology Network [Internet]. Oxford: Malaria Genomic Epidemiology Network; c2018 [cited 2017 Oct 11]. People; [about 3 screens]. Available from: <https://www.malariagen.net/network/people>.
25. Malaria Genomic Epidemiology Network. Data Release Policy for Genome-Wide Association Data. Oxford: Malaria Genomic Epidemiology Network; 2008.
26. Malaria Genomic Epidemiology Network [Internet]. Oxford: Malaria Genomic Epidemiology Network; c2018 [cited 2017 Oct 11]. Human GWAS Data Terms of Use; [about 3 screens]. Available from: <https://www.malariagen.net/data/terms-use/human-gwas-data>.
27. Malaria Genomic Epidemiology Network. Joint Policy on Data Sharing, Intellectual Property and Publications. Oxford: Malaria Genomic Epidemiology Network; 2008.
28. Wellcome Trust. Sharing Data from Large-scale Biological Research Projects: A System of Tripartite Responsibility. London: Wellcome Trust; 2003.
29. Chotski DA, Parker M, Kwiatkowski DP. Data sharing and intellectual property in a genomic epidemiology network. Bull World Health Organ. 2006;84:382-387.
30. Parker M, Bull SJ, De Vries J, Agbenyega T, Doumbo OK, Kwiatkowski DP. Ethical Data Release in Genome-Wide Association Studies in Developing Countries. PLoS Med 2009;6:e1000143.
31. Malaria Genomic Epidemiology Network [Internet]. Oxford: Malaria Genomic Epidemiology Network; c2018 [cited 2017 Oct 11]. *P. Falciparum* Community Project Terms of Use; [about 3 screens]. Available from: <https://www.malariagen.net/data/terms-use/p-falciparum-community-project-terms-use>.
32. Malaria Genomic Epidemiology Network [Internet]. Oxford: Malaria Genomic Epidemiology Network; c2018 [cited 2017 Oct 11]. Pf3k Pilot Phase Terms of Use; [about 2 screens]. Available from: <https://www.malariagen.net/data/terms-use/pf3k-pilot-phase-terms-use>.
33. Malaria Genomic Epidemiology Network [Internet]. Oxford: Malaria Genomic Epidemiology Network; c2018 [cited 2017 Oct 11]. Ag1000g Terms of Use; [about 2 screens]. Available from: <https://www.malariagen.net/data/terms-use/ag1000g-terms-use>.
34. Malaria Genomic Epidemiology Network [Internet]. Oxford: Malaria Genomic Epidemiology Network; c2018 [cited 2017 Oct 11]. Coordination; [about 1 screen]. Available from: <https://www.malariagen.net/about/coordination>.
35. Malaria Genomic Epidemiology Network [Internet]. Oxford: Malaria Genomic Epidemiology Network; c2018 [cited 2017 Oct 11]. Capacity building; [about 2 screens]. Available from: <https://www.malariagen.net/network/capacity-building>.
36. Malaria Genomic Epidemiology Network [Internet]. Oxford: Malaria Genomic Epidemiology Network; c2018 [cited 2017 Oct 11]. Governance Committee; [about 2 screens]. Available from: <https://www.malariagen.net/projects/consortial-project-1/governance-committee>.
37. Malaria Genomic Epidemiology Network [Internet]. Oxford: Malaria Genomic Epidemiology Network; c2018 [cited 2017 Oct 11]. Data Bursary Scheme; [about 3 screens]. Available from: <https://www.malariagen.net/network/capacity-building/data-bursary-scheme>.
38. Malaria Genomic Epidemiology Network [Internet]. Oxford: Malaria Genomic Epidemiology Network; c2018 [cited 2017 Oct 11]. Courses and workshops; [about 3 screens]. Available from: <https://www.malariagen.net/network/capacity-building/courses-and-workshops%20>.
39. Parker M, Kwiatkowski DP. The ethics of sustainable genomic research in Africa. Genome Biol. 2016;17:44.
40. De Vries J, Bull SJ, Doumbo O, Ibrahim M, Mercereau-Puijalon O, Kwiatkowski D, et al. Ethical issues in human genomics research in developing countries. BMC Med Ethics. 2011;12:5.
41. Malaria Genomic Epidemiology Network. Data Access Agreement. Oxford: Malaria Genomic Epidemiology Network; 2015.
42. Structural Genomics Consortium [Internet]. Toronto: Structural Genomics Consortium; [cited 2017 Oct 11]. Structural Parasitology; [about 3 screens]. Available from: <http://www.thesgc.org/science/parasitology>.
43. Structural Genomics Consortium [Internet]. Toronto: Structural Genomics Consortium; [cited 2017 Oct 11]. Structure-guided Drug Discovery Coalition; [about 5 screens]. Available from: <http://www.thesgc.org/sddc>.
44. Structural Genomics Consortium [Internet]. Toronto: Structural Genomics Consortium; [cited 2017 Oct 11]. People; [about 3 screens]. Available from: <http://www.thesgc.org/people>.
45. Structural Genomics Consortium [Internet]. Toronto: Structural Genomics Consortium; [cited 2017 Oct 11]. Partners; [about 5 screens]. Available from: <http://www.thesgc.org/about/partners>.
46. Structural Genomics Consortium [Internet]. Toronto: Structural Genomics Consortium; [cited 2017 Oct 11]. Academic Collaborators; [about 2 screens]. Available from: <http://www.thesgc.org/people/academic-collaborators>.
47. Structural Genomics Consortium [Internet]. Toronto: Structural Genomics Consortium; [cited 2017 Oct 11]. Industrial/SME Collaborators; [about 2 screens]. Available from: <http://www.thesgc.org/people/industrial-sme-collaborators>.
48. Structural Genomics Consortium [Internet]. Toronto: Structural Genomics Consortium; [cited 2017 Oct 11]. FAQ for Non-Scientists; [about 5 screens]. Available from: <http://www.thesgc.org/about/mini_faq>.
49. Structural Genomics Consortium [Internet]. Toronto: Structural Genomics Consortium; [cited 2017 Oct 11]. Governance; [about 5 screens]. Available from: <http://www.thesgc.org/about/governance>.
50. Structural Genomics Consortium [Internet]. Toronto: Structural Genomics Consortium; [cited 2017 Oct 11]. CREATE ChemNET: A Medicinal Chemistry Network in Epigenetics; [about 3 screens]. Available from: <http://www.thesgc.org/chemnet>.
51. Structural Genomics Consortium [Internet]. Toronto: Structural Genomics Consortium; [cited 2017 Oct 11]. Symposia; [about 3 screens]. Available from: <http://www.thesgc.org/events/symposia>.
52. Edwards AM. Open access chemical and clinical probes to support drug discovery. Nat Chem Biol. 2009;5:436-440.
53. Edwards A. Why put science in the public domain?. 2013 August 2 [cited 11 October 2017]. In: SGC in the open [Internet]. Toronto: Structural Genomics Consortium. [about 3 screens]. Available from: <http://www.thesgc.org/blog/why-put-science-in-the-public-domain>.
